# Modulatory Effect of Probiotic *Lactobacillus rhamnosus* PB01 on Mechanical Sensitivity in a Female Diet-Induced Obesity Model

**DOI:** 10.1155/2021/5563959

**Published:** 2021-06-29

**Authors:** Hiva Alipour, Parisa Gazerani, Mahmoud Heidari, Fereshteh Dardmeh

**Affiliations:** ^1^Department of Health Science and Technology, Faculty of Medicine, Aalborg University, Aalborg, Denmark; ^2^Department of Life Sciences and Health, Faculty of Health Sciences, Oslo Metropolitan University, Oslo, Norway; ^3^Department of Biology, Islamic Azad University, Gorgan Branch, Gorgan, Iran

## Abstract

Obese animals and humans demonstrate higher sensitivity to pain stimuli. Among the endogenous factors prompting obesity, the intestinal microbiota has been proposed to influence responsiveness to pain. The beneficial effects of probiotics on obesity are well documented, whereas data on their analgesic efficacy is minimal. The protective effect of probiotics on nociception in diet-induced obese male mice has been previously demonstrated, but the sex differences in pain sensitivity and analgesic response do not allow for the generalization of these findings to the female gender. Hence, this study aimed at investigating the potential effects of oral probiotic supplementation on mechanical pain thresholds in female diet-induced obese mice compared with controls. Thirty-two adult female mice (*N*=32) were randomly divided into two groups receiving standard (normal-weight group; NW) or high-fat diet (diet-induced obesity; DIO). All rats received a single daily dose (1 × 10^9^ CFU) of probiotics (*Lactobacillus rhamnosus* PB01, DSM14870) for four weeks by gavage. Mechanical pain thresholds were recorded by an electronic von Frey device at baseline, at the end of weeks 2, 4, 6, and 8 in both DIO and NW groups with and without consumption of probiotics. Blood samples were obtained for the measurement of lipid profile and reproductive hormone levels. Bodyweight was considerably lower (*P* < 0.001) in groups supplied with probiotics than groups without probiotics. Pressure pain threshold values showed a significant (*P* < 0.001) increase (reduced pain sensitivity) following probiotic supplementation, proposing a modulatory effect of probiotics on mechanical sensory circuits and mechanical sensitivity, which might be a direct consequence of weight loss or an indirect result of the probiotics' anti-inflammatory properties. Understanding the precise underlying mechanism for the effect of probiotics on weight loss and mechanical pain sensitivity seen in this study warrants further investigation.

## 1. Introduction

Pain is a complex, multidimensional perception that varies in quality, strength, duration, location, and unpleasantness. The strength and unpleasantness of pain are neither simply nor directly related to the nature and extent of tissue damage [1]. Physiological pain is a warning mechanism that protects an organism by inducing a withdrawal response to harmful stimuli, while chronic pain indicates medical pathogenesis that needs treatment, which is often challenging [2]. Neurotransmitters, immune cells, and hormones have been demonstrated to contribute to the pathogenesis of chronic pain [3].

Biological factors, including gender and genetics, have been shown to alter pain perception both in humans and animals [4]. The influence of diet on pain perception is also well known [5]. It has been suggested that adiposity is strongly associated with pain, which is more prevalent in obese than nonobese individuals [6]. Obesity alters adipose tissue metabolic and endocrine functions, which have been previously reported to influence pain perception [6]. The adipose tissue generates proinflammatory cytokines such as interleukin 6 (IL-6), tumor necrosis factor *α* (TNF-*α*), and leptin, in addition to C-reactive protein (CRP) which is released from the liver in response to IL-6 [7].

Studying the association between pain and obesity in patients is somewhat challenging; this is mainly due to the multifaceted and subjective nature of pain, the complexity of obesity, and its related confounding factors [8]. Mouse models of obesity, in which responsiveness to controlled pain stimuli can be assessed, provide a control platform to study coexisting conditions of pain and obesity and the effects of modulating strategies, such as dietary factors. The gut microbiota composition has been implicated in obesity development [9]. This composition can be influenced by many factors such as medicine, disease, host genetics, and diet, which is considered the major contributor [10]. Many studies have indicated that diet can alter the ratio of two critical bacterial divisions known as Bacteroidetes and Firmicutes. These studies have reported exposure to a high-fat diet to decrease Bacteroidetes and increase Firmicutes phyla levels [11].

Furthermore, losing weight by following a low-calorie diet may increase the abundance of Bacteroidetes in obese people [12]. Thereby, modulation of the gut microbiota using probiotic supplements may offer a novel tool in obesity management. Probiotics have shown antiobesity properties, anti-inflammatory/antioxidant properties, and the ability to modify energy homeostasis and enhance gut and systemic immune function [13]. On the other hand, probiotics can act on the intestinal tract and protect it from other microbes and pathogens either by competing with them for receptors and binding sites, thus preventing the microbes from adhering to the intestinal mucus [14], by strengthening the gut epithelial barrier [15], or by producing antimicrobial agents to suppress another microorganisms' growth [16]. Probiotics have also shown a beneficial effect on gastrointestinal-related pain. For example, a study on irritable bowel syndrome patients treated with *L. plantarum* showed decreased pain sensitivity in these patients [17]. However, studies focusing on other types of pain are lacking.

A preclinical study demonstrated that diet-induced obese (DIO) male mice receiving oral probiotic supplementation demonstrated lower sensitivity to pressure pain than DIO male mice without probiotic supplementation [4]. However, a growing body of evidence confirms sex differences in pain sensitivity and analgesic response, observed in acute and chronic clinical pain and experimental models [18]. Overall, females have higher pain sensitivity to several pain stimuli (e.g., mechanical, thermal, chemical, and electrical) and a higher prevalence of chronic pain conditions such as fibromyalgia, temporomandibular disorders, or headaches [19].

Females are also reported to have a higher prevalence of obesity [20]. Thus, the results of the above-mentioned study on male mice [4] cannot be generalized to the female.

There are limited studies on the underlying mechanisms of pain-obesity focusing on the female population, while the effects of dietary components and supplementation with probiotics on pain sensitivity in female mouse models have not yet been investigated.

Therefore, this study aimed to assess the impact of oral administration of *Lactobacillus rhamnosus PB01* (DSM 14870) on pressure pain thresholds in normal-weight and diet-induced obese female mouse models to address if probiotic supplements can potentially overcome the deleterious effects of obesity and reduce pain sensitivity. The results of this study would also provide evidence on whether consumption of probiotics can modulate pain, obesity, or both and if hormonal changes would be present in response to the supplementation.

## 2. Materials and Methods

All experiments were carried out following the Guidelines for Animal Experimentation and Approval of “The Danish Animal Experiments Inspectorate” (study case number: 2016−15−0201−00867). This study was performed on thirty-two adult female C57BL/6NTac mice (Taconic, Denmark), housed in a room with 60% humidity, 22°C to 24°C temperature, and 12 h dark-light cycles (light on from 0800 to 2000 h). Mice were allowed two weeks of adaptation and free access to their respective diets and tap water ad libitum during the study period.

### 2.1. Study Design

The study consisted of two phases.

Phase I: following the adaptation period, the estrus cycles were synchronized before randomly dividing the mice into two groups to be fed for four weeks on a high-fat (60%) Research Rodent Diet (D12492, Research Diets, Inc., USA) to create the diet-induced obesity (DIO) model or a standard diet (normal-weight group; NW) as the control (lean) group.

Phase II: after four weeks on the respective diets, each group was further divided into two subgroups. The four new groups continued with the previous diet with or without probiotic supplements for four weeks, creating the following four diet groups.

  Group 1: lean group on normal diet (ND)
  Group 2: lean group on normal diet and probiotic supplement (NDPR)
  Group 3: DIO group on fat diet (FD)
  Group 4: DIO on fat diet and probiotic supplement (FDPR)

### 2.2. Estrus Cycle Synchronization

After the adaptation phase, to synchronize the estrous cycles, the mice were given a single intraperitoneal injection of 0.5 *μ*g of coprostanol (CC-13104; Cayman Chemical, USA) and 3 *μ*g of subcutaneous progesterone (Cayman Chemical, USA), followed by 0.5 *μ*g of coprostanol three days later [21]. During weeks 3-4 and 7-8 of the study, animals were checked for the estrus cycle by examining vaginal cytology and allowed at least one regular estrous cycle (4-5 days in length) before blood collection during the diestrus phase. The mice were put on the research diet (according to the diet groups explained earlier) one day after the estrus synchronization.

### 2.3. BMI Measurements

BMI of the mice was calculated once every two weeks by measuring the weight and the length of the animals from the tip of the nose to the end of the tail.

### 2.4. Probiotics

*Lactobacillus rhamnosus PB01*, *DSM 14870*, was provided as a lyophilized powder by Deerland Probiotics and Enzymes (Hundested, Denmark). Aliquots providing 1 × 10^9^ CFU per mouse were prepared (based on the manufacturer's guidelines) and stored at −20°C until use. The prepared probiotic aliquots were diluted with normal saline at room temperature shortly before use (0.25 ml per mouse) and given to the NDPR and FDPR groups orally by a gavage needle. ND and FD received normal saline without probiotics. This process was repeated once daily during phase II (second 4 weeks) of the study.

### 2.5. Pressure Pain Threshold

Pressure pain threshold (PPT) is defined as the minimum force applied, which induces pain. This measure has proven to be commonly useful in evaluating multiple clinical pain states [22, 23]. Rodent withdrawal reflex to pressure application upon the sensation of pain is interpreted as similar to PPT assessment in humans. In this study, mechanical pressure was applied to the animal paw using the electronic Von Frey device (Bioseb, France), and the pressure at which the paw withdrawal happened was recorded. Mechanical sensitivities reflected on PPT values were obtained every two weeks to estimate nociception levels concerning obesity and probiotics administration. Faster withdrawal with lower PPT values was considered a higher sensitivity to mechanical stimuli.

### 2.6. Blood Serum Collection and ELISA Tests

Blood samples were collected at the beginning of the study (baseline, after synchronization), and every two weeks throughout the study (at the end of weeks 2, 4, 6, and 8) from the facial vein of conscious mice [24].

Blood serum was immediately collected by centrifugation (500 g for 10 min at 4°C) and stored at −20°C until the assessment of blood lipid profiles (total cholesterol (TC), high-density lipoprotein (HDL), and low/very low-density lipoprotein (LDL/VLDL)) using a commercially available Elisa assay kit (ab65390, Abcam, United Kingdom), according to the manufacturer's directions. Blood serum FSH, LH, testosterone, and leptin levels were also measured using commercially available ELISA kits (MyBioSource, USA; Cat. No: MBS703380, MBS041300, MBS7606180, and MBS2885529, resp.) according to the manufacturer's directions.

### 2.7. Statistical Analysis

The results have been presented as means ± standard deviation (SD) unless stated otherwise. The Shapiro–Wilk test was used to confirm the normal distribution of the data. The repeated measures analysis of variance (ANOVA) was used to compare differences in pain sensitivity, weight, lipid profiles, and hormone levels between groups. Tukey's multiple comparison tests were used for pairwise comparison of the hormone levels between the groups. GraphPad Prism version 8.0.0 (224) was used to perform the statistical analysis and *P* < 0.05 was considered significant.

## 3. Results

### 3.1. The Effect of Probiotic Supplementation on Total Body Weight

**Figure 1:**
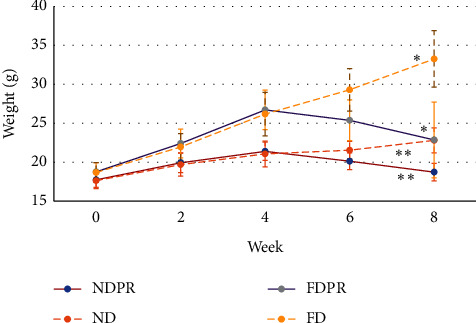
Estimated marginal means of weight in mice on normal diet (ND), fat diet (FD), normal diet with probiotic supplementation (NDPR), and fat diet with probiotic supplementation (FDPR) at weeks 0 (baseline), 2, 4, 6, and 8 of the study. Asterisks mark pairwise significance (*P* > 0.05).

As illustrated in Figure 1, all groups demonstrated weight gain during phase I (the first four weeks). This increase was significantly higher in the FD compared to the ND group (*P* < 0.0001).

During phase II, the FD and ND groups maintained a rising trend, whereas the FDPR and NDPR groups which received *Lactobacillus rhamnosus* (PB01, DSM 14870) showed a decrease in weight gain. At the end of the study, the FDPR and NDPR groups presented a significantly lower weight than the FD and ND groups, respectively (*P* < 0.001). At the end of week 8, the FD group demonstrated a significantly higher weight compared to the ND group (*P* < 0.001), while the NDPR group demonstrated a significantly lower weight compared to the ND group (*P* < 0.001).

### 3.2. The Effect of Probiotic Supplementation on Pressure Pain Threshold

**Figure 2:**
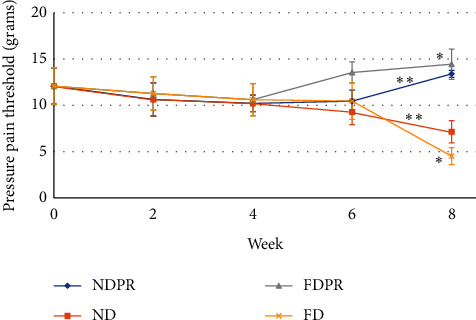
Estimated marginal means of pressure pain threshold (PPT) in mice on normal diet (ND), fat diet (FD), normal diet with probiotic supplementation (NDPR), and fat diet with probiotic supplementation (FDPR) at weeks 0 (baseline), 2, 4, 6, and 8 of the study. Asterisks mark pairwise significance (*P* > 0.05).

Figure 2 illustrates the mean PPT values in the different groups throughout the study. From weeks 0 to 4 (phase I), mice in both ND and FD groups without probiotics supplementation demonstrated gradually lower PPT values (higher pain sensitivity). From weeks 4 to 8 (during phase II), the ND and FD groups continued lowering values in PPT (higher pain sensitivity), with a larger decrease in FD compared to ND (*P* < 0.001).

From weeks 4 to week 6, the NDPR group demonstrated a slight increase in PPT (less sensitivity), followed by a sudden significant increase from week 6 to week 8. Mice on the fat diet with probiotic supplementation (FDPR) showed an increase in PPT from week 4 to week 6 (less sensitivity) (*P* < 0.001), continuing to significantly higher values compared to NDPR at week 8 (*P* < 0.001).

### 3.3. The Effect of Probiotic Supplementation on Lipid Profiles (Total Cholesterol, LDL/VLDL, HDL)

The lipid profiles (total cholesterol, LDL/VLDL, and HDL levels) in mice of the different groups (ND, NDPR, FD, and FDPR) at the end of the study are illustrated in Table 1.

Total cholesterol showed significantly higher levels in the FD group compared to ND at the end of the study (week 8; *p* < 0.05). LDL/VLDL and HDL also showed higher tendency levels in the FD group compared to ND at the end of the study.

ND and FD groups also demonstrated higher tendency levels of TC compared to NDPR and FDPR, respectively. LDL levels showed a lower trend in FDPR than FD, and HDL levels demonstrated a lower tendency in NDPR than ND.

### 3.4. The Effect of Probiotic Supplementation on Sex Hormone Levels (Testosterone-FSH-LH)

The blood testosterone, FSH, and LH levels in different groups of mice (ND, NDPR, FD, and FDPR) at the end of the study are illustrated in Table 2. NDPR and FDPR demonstrated higher testosterone levels than ND and FD groups, respectively, at the end of the study; however, the difference remained insignificant. There was no significant difference between ND and FD groups during phase I (from baseline to week 4). LH concentrations were similar among ND, FD, and NDPR. The FDPR group had lower LH levels than the FD group; however, the difference remained insignificant.

FSH concentrations were significantly higher in the NDPR group compared to ND (*p* < 0.05). However, there was no significant difference when comparing FDPR to FD and FD to ND, although a trend was observed.

### 3.5. The Effect of Probiotic Supplementation on Leptin Levels

The effect of probiotic supplementation on leptin levels is illustrated in Table 2. Leptin concentrations showed significantly (*P* < 0.001) higher levels in the FD group than the ND group and significantly higher levels in the FDPR group than the NDPR group.

Leptin concentrations were higher in the ND group than the NDPR group and lower in the FD group than the FDPR group with no significant difference.

### 3.6. Figures, Tables, and Schemes

Estimated marginal means of weight and pressure pain threshold are shown in Figures 1 and 2. Mean (±standard deviation) concentration of serum lipid profiles and testosterone, LH, and FSH levels are provided in Tables 1 and 2.

## 4. Discussion

### 4.1. Probiotic Supplementation and Body Weight

The results of this study showed that probiotics could influence body weight and reduce it. During phase I (before receiving probiotics, baseline to week 4), all groups demonstrated weight gain, whereas, during phase II (weeks 4–8), groups that received probiotics (NDPR, FDPR) demonstrated weight loss, whereas groups without probiotics' consumption (ND, FD) continued gaining weight until the end of the study. These results align with a previous study demonstrating the weight loss effects of probiotics in humans with similar probiotic strains [25]. *Lactobacillus rhamnosus GGMCC* reviewed in another article also demonstrated a positive effect on weight loss in animals and humans [26].

The possible underlying mechanisms of action of probiotics on body weight have been described in several studies. One study described the ability of the probiotic (*Lactobacillus rhamnosus* GG (LGG)) to reduce gaining weight in mice fed with a high-fat diet by regulating lipid and glucose metabolism [27]. It was reported that *LGG* sensitizes insulin action by enhancing adiponectin production in white adipose tissue, AMP-activated protein kinase (AMPK) activation, and increasing the expressions of GLUT4 and lipid oxidative genes in adipose tissue [27]. *Lactobacillus rhamnosus* PL60 has shown a significant reduction in white adipose tissue by producing conjugated linoleic acid in mice fed with diet-induced obesity [28]. *Lactobacillus Plantarum* FH185 is suggested to potentially reduce adipocyte size by preventing adipocyte differentiation and inhibiting lipase activity [29], hence demonstrating antiobesity properties.

Dardmeh et al. previously demonstrated that male mice on a high-fat diet supplemented with *Lactobacillus rhamnosus* PB01 (DSM 14870) maintained a stable weight, while the same diet without probiotic supplementation led to a massive weight gain [4, 30]. This study's results combined with those previously reported by Dardmeh et al. [4] demonstrated that probiotics affect weight sex independently, possibly indicating that hormonal levels might not be playing a role as prominent as other proposed mechanisms of action for the probiotics' weight-management properties.

The amount of consumed food and type and quantity of faeces among different groups was not considered objectively in this study. Comparative assessment of food consumption, faeces type, and bacterial and archaeal community analysis (16 s) of the gut microbiota in the different groups by future studies, could provide an insight into the underlying mechanism of the weight- and pain-reducing effect of the probiotics.

### 4.2. Probiotics Supplementation and Pressure Pain Threshold

Our results showed a higher pain sensitivity (reflected by lower PPT values) in the FD than the ND group, which was not significant, possibly due to the small sample size or variations in the withdrawal measurement method. Nevertheless, these results support the findings of several previous clinical studies, where the presence of pain complaints showed to be more common in people with high BMI compared to people with normal or low BMI [31–33]. Furthermore, obese adults and children are reported to exhibit more pain complaints [34, 35]. However, there is still some controversy in the literature. A study carried out by Zahorska-Markiewicz et al. demonstrated that weight-reducing treatment did not change the pain sensitivity in obese women [36]. Moreover, obese rats and obese people are less sensitive to pain stimuli than nonobese controls [37].

Tashani et al. conducted a study to investigate the relation between the percentage and distribution of fat with pain sensitivity response, reporting that the body site and the percentage of subcutaneous fat might affect the pain response to different types and intensities of stimuli. Furthermore, they found that obese individuals were more sensitive than nonobese people in response to pressure pain, but not thermal pain [38].

Biomechanical factors and chemical mediators have been considered as major underlying factors of the obesity-pain association. High weight in obese individuals increases the pressure on joints and results in defective structural changes [39]. Furthermore, since adipocytes release proinflammatory markers (TNF-a, IL-6, and CRP) [40], obesity may dysregulate the inflammatory markers and potentiate the inflammatory response, which could lead to higher pain sensitivity [7]. Ianniti et al. found that obese mice exhibited more significant peripheral inflammation than lean mice using carrageenan injection in the paw [41].

Our results also found that oral probiotics supplementation increased PPT values (lower mechanical pain sensitivity). This result agrees with previous studies, showing increased PPT in male mice supplemented with probiotics than groups without probiotics consumption [4].

Mechanisms underlying the reduction of pain sensitivity following the administration of probiotics are not well investigated. A few studies have suggested that probiotic strains display anti-inflammatory effects by downregulating the production of inflammatory cytokines, thus controlling pain [42]. A study performed by Abdelouhab et al. investigated the anti-inflammatory effect of “Ultrabiotique^®^” (a probiotic) in ulcerative colitis (UC) treatment. Oral administration of “Ultrabiotique^®^” decreased nitric oxide levels, which usually rise in UC disease, which could confirm the anti-inflammatory properties of probiotics [43]. Further investigation to point out the precise mechanism is warranted.

### 4.3. Probiotic Supplementation and Serum Lipid Profile (Total Cholesterol, LDL/VLDL, HDL)

Total cholesterol levels were significantly higher in the FD group compared to ND. Some previous studies have also reported that people with increased BMIs showed higher serum total cholesterol than normal-weight individuals [20, 44].

Probiotics supplements demonstrated a trend towards reduced total cholesterol in both ND and FD groups and a tendency towards reduced LDL/VLDL in the FD group, which are in line with the direction of the findings in some previous studies [4, 45]. It is possible that the dose or duration of probiotic supplementation can influence the outcome. Combinations of different strains of probiotics can also be a choice.

The study implemented by Guo et al. showed that a diet rich in probiotics decreased TC and LDL concentrations in individuals with high and normal cholesterol levels (Guo et al., 2011).

A recent study suggested that probiotics have beneficial effects on bile salt hydrolase (BSH) activity, which plays a role in lipase action. Higher lipase activity accelerates the breakdown of fat, thus decreasing body weight and plasma cholesterol levels [46].

### 4.4. Probiotic Supplementation and Sex Hormone Levels (Testosterone-FSH-LH)

No significant difference was observed in testosterone concentrations between ND and FD groups in this study; however, the observed trends towards higher levels of testosterone in the NDPR and FDPR compared to ND and FD groups are in line with the findings of a previous study where another strain of *Lactobacillus* (*L. reuteri*) significantly increased testosterone concentration in mice regardless of the type of the diet [47, 48]. It has to be noted that the different genders, supplementation periods, doses, and strains of probiotics used in different studies make comparisons between studies rather complicated and challenging.

In groups without probiotic supplementation, FSH showed a tendency towards higher levels in the FD compared to the ND group. In the probiotic supplemented groups, the lean mice showed a significant increase in FSH levels, with a similar trend observed in the diet-induced obese mice. Similar previous studies have also described an increase in FSH due to probiotic supplementation [49].

The increase in testosterone and FSH levels following probiotics supplementation may be explained by the relation between obesity and testosterone, in which obesity decreases testosterone levels. Weight loss can also decrease the proinflammatory cytokines and CRP, suppressing their effect on the hypothalamic-pituitary axis (HPA), followed by negative feedback increasing GnRH secretion and testosterone levels [50]. Lack of change in LH between the groups could have been due to the estrous cycle synchronization, making it challenging to see small changes in hormone levels [51].

### 4.5. Probiotic Supplementation Effects on Leptin Levels

Leptin is known to increase in people with obesity as it is produced by adipose tissue, and the circulating concentration of leptin is positively affected by body fat stores [52]. The FDPR group demonstrated a tendency towards higher levels of leptin compared to the FD group. The increased leptin levels send signals to the hypothalamic receptors to inhibit appetite and stimulate metabolic rate and thermogenesis [53]. The normal-diet group with probiotic supplementation (NDPR) demonstrated lower leptin levels than the ND group, although not significantly. The decreased concentrations of leptin correlate with the lower weight of mice in the NDPR group.

## 5. Conclusions

Overall, this study provided evidence that *Lactobacillus rhamnosus* (PB01, DSM 14870) probiotic supplementation reduces weight and pain sensitivity in a female diet-induced obesity mouse model, although the underlying mechanisms remain to be investigated. This study, combined with previous reports, indicates that the effect of probiotics on weight is sex-independent, suggesting that hormonal levels might not play a prominent role in the weight-management pain-sensitivity lowering properties of probiotics. Regardless of the underlying mechanism, *Lactobacillus rhamnosus* (PB01, DSM 14870) supplementation can be proposed as a candidate for an innovative weight and pain management strategy in both obese or normal-weight females.

Translation of this result to humans may lead to a novel therapeutic approach to pain management of obese or normal-weight individuals in the future.

## Figures and Tables

**Table 1:** Mean (±standard deviation) concentration of serum lipid profiles in mice on normal (ND), high-fat diet (FD), normal diet with probiotics (NDPR), or fat diet with probiotics (FDPR) at the end of the study (week 8). Similar letters demonstrate significant between-group differences (*P* < 0.05).

Serum lipid profile	Group			
	ND	NDPR	FD	FDPR
LDL/VLDL (*μ*g/*μ*l)	47.05 ± 11.76	52.45 ± 9.04	58.18 ± 16.73	55.06 ± 15.73
Total cholesterol (*μ*g/*μ*l)	76.88 ± 6.72^a^	76.72 ± 8.09	90.82 ± 5.62^a^	87.33 ± 6.393
HDL (*μ*g/*μ*l)	29.83 ± 12.64	24.27 ± 4.85	32.64 ± 17.64	32.27 ± 15.4

**Table 2:** Mean (±standard deviation) concentration values of testosterone, LH, and FSH levels in mice on normal (ND), high-fat diet (FD), normal diet with probiotics (NDPR), or fat diet with probiotics (FDPR) at the end of the study (week 8). Similar letters demonstrate significant between-group differences (*P* < 0.05).

Hormone	Group			
	ND	NDPR	FD	FDPR
Testosterone (ng/dl)	10.25 ± 3.59	13.35 ± 4.51	10.27 ± 2.77	13.04 ± 3.8
LH (mIU/ml)	5.32 ± 0.74	5.26 ± 0.62	5.22 ± 0.69	5.02 ± 1.04
FSH (mIU/ml)	29.49 ± 9.41^a^	40.84 ± 3.81^a^	37.94 ± 3.68	44.94 ± 6.49
Leptin (ng/ml)	1.15 ± 0.2^b^	1.09 ± 0.08	2.099 ± 0.21^b^	2.20 ± 0.15

## Data Availability

The data can be accessed upon request from the corresponding author (feda@hst.aau.dk).
